# Potential for broad-scale transmission of Ebola virus disease during the West Africa crisis: lessons for the Global Health security agenda

**DOI:** 10.1186/s40249-017-0373-4

**Published:** 2017-12-01

**Authors:** Eduardo A. Undurraga, Cristina Carias, Martin I. Meltzer, Emily B. Kahn

**Affiliations:** 1 National Center for Emerging and Zoonotic Infectious Diseases, Centers for Disease Control and Prevention, Atlanta, Georgia USA; 2 Present address: School of Government, Pontificia Universidad Católica de Chile, Santiago, Región Metropolitana Chile

**Keywords:** Ebola virus, Global health, Transmission, Epidemics, Viruses, Communicable diseases, Western Africa, Guinea, Liberia, Sierra Leone

## Abstract

**Background:**

The 2014–2016 Ebola crisis in West Africa had approximately eight times as many reported deaths as the sum of all previous Ebola outbreaks. The outbreak magnitude and occurrence of multiple Ebola cases in at least seven countries beyond Liberia, Sierra Leone, and Guinea, hinted at the possibility of broad-scale transmission of Ebola.

**Main text:**

Using a modeling tool developed by the US Centers for Disease Control and Prevention during the Ebola outbreak, we estimated the number of Ebola cases that might have occurred had the disease spread beyond the three countries in West Africa to cities in other countries at high risk for disease transmission (based on late 2014 air travel patterns). We estimated Ebola cases in three scenarios: a delayed response, a Liberia-like response, and a fast response scenario. Based on our estimates of the number of Ebola cases that could have occurred had Ebola spread to other countries beyond the West African foci, we emphasize the need for improved levels of preparedness and response to public health threats, which is the goal of the Global Health Security Agenda. Our estimates suggest that Ebola could have potentially spread widely beyond the West Africa foci, had local and international health workers and organizations not committed to a major response effort. Our results underscore the importance of rapid detection and initiation of an effective, organized response, and the challenges faced by countries with limited public health systems. Actionable lessons for strengthening local public health systems in countries at high risk of disease transmission include increasing health personnel, bolstering primary and critical healthcare facilities, developing public health infrastructure (e.g. laboratory capacity), and improving disease surveillance. With stronger local public health systems infectious disease outbreaks would still occur, but their rapid escalation would be considerably less likely, minimizing the impact of public health threats such as Ebola.

**Conclusions:**

The Ebola outbreak could have potentially spread to other countries, where limited public health surveillance and response capabilities may have resulted in additional foci. Health security requires robust local health systems that can rapidly detect and effectively respond to an infectious disease outbreak.

**Electronic supplementary material:**

The online version of this article (10.1186/s40249-017-0373-4) contains supplementary material, which is available to authorized users.

## Multilingual abstracts

Please see Additional file 1 for translation of the abstract into the six official working languages of the United Nations.

## Background

The 2014–2016 Ebola crisis in West Africa was unprecedented in scale, with about 28652 suspected, probable, or confirmed Ebola cases (15261 cases were laboratory confirmed), and 11,325 deaths [1, 2]. All previous outbreaks combined (1976–2008) resulted in 2232 reported Ebola cases and 1503 reported deaths [2]. The response effort by local and international health workers and organizations resulted in control of the Ebola outbreak in West Africa by the end of 2015, but not without significant costs for both donor and afflicted countries [3]. By December 2015, the response had cost donors at least US$ 3.6 billion [2]; an additional estimated US$ 2.2 billion in gross domestic product (GDP) were lost in Guinea, Liberia, and Sierra Leone in 2015. More than 17,300 children lost one or both parents to Ebola [2]. Furthermore, access to healthcare services decreased substantially, resulting in a considerable hidden morbidity and mortality burden [4, 5].

The 2014–2016 Ebola crisis highlighted the need for a coherent, effective, predictable, and organized global response system to public health emergencies [6]. The crisis also confirmed that few countries had complied with International Health Regulations (IHR), which require countries to build capabilities to detect and respond to potential public health emergencies of international concern [6–8]. Many countries had invested only limited resources in health infrastructure, including human resource capabilities, lab infrastructure, and disease surveillance [9]. Consequently, it would have been difficult for local and international organizations to respond to potential active Ebola virus transmission beyond West Africa. Even with a geographically limited outbreak, delays in response efforts resulted in a higher number of Ebola cases at peak [2, 10, 11].

The introduction of multiple Ebola cases to some of the world’s poorest countries with limited early detection and response capabilities and healthcare infrastructure [3, 12–16] highlighted the risk of widespread transmission. On March 23 2014, Guinea reported the first cases of the West African Ebola outbreak to the World Health Organization (WHO) [17]. By May 2014, the first Ebola cases were reported in both Liberia and Sierra Leone [2, 15]. In August, WHO declared the Ebola epidemic to be a Public Health Emergency of International Concern with a “vastly underestimated” death toll of approximately 1000 persons [6]. Ebola cases also occurred in Nigeria, Mali, and Senegal, but were detected and controlled due to efforts from local and international health workers and organizations [3, 15, 17–20]. Ebola cases were also treated in Italy, Spain, the United Kingdom, and United States. These were mostly healthcare workers who had contracted the disease in West Africa [2]. The risk of Ebola- infected persons traveling to other countries has been highlighted by mathematical models [15, 19, 21–28]. The biggest risk, however, was in the ability to respond to any additional cases and outbreaks occurring outside the West African foci. The US Centers for Disease Control and Prevention (CDC), for example, characterized their response as “the most intensive outbreak response in the agency’s history” [2]. It is reasonable to question how much additional capacity existed among international health agencies to aid national health agencies with limited resources in a rapid response to yet another Ebola outbreak.

Here we present estimates of the number of cases that might have occurred had Ebola been introduced from the three Ebola-affected countries (Liberia, Sierra Leone and Guinea) and started to spread to cities in other low- and middle-income countries, based on air travel patterns in late 2014 [10]. These cities typically have limited local public health resources and emergency response capabilities. We assumed that some number of Ebola cases would occur in a country before the outbreak is detected and an effective response is initiated, and that responding to the foci in West Africa would have limited the ability of international public health agencies to rapidly aid the responses to additional Ebola outbreaks. Our estimates emphasize the need for improved levels of preparedness and response to public health threats, underscore the importance of rapid detection and initiation of an effective, organized response, and highlight some of the challenges faced by countries with limited public health systems that drove the progression of the Ebola outbreak. Based on these lessons, we produced a list of actionable items that would contribute to a rapid detection and effective responses in countries at high risk for disease transmission, thus minimizing the impact of emergence or re-emergence of infectious disease threats.

## Main text

### Methods and main illustrative estimates: Potential Ebola broad-scale transmission scenario

We used the *EbolaResponse* spreadsheet-based model developed during the early stages of the response [10] to estimate, in three scenarios of disease detection and control, how many cases might have occurred had Ebola spread from the three affected countries (Liberia, Sierra Leone and Guinea) to cities in other low- and middle-income countries, given usual air-travel patterns [29] (Additional file 2). The model, a Markov Chain, tracks patients through the following states: susceptible to disease (S), infected people incubating Ebola virus (I), infectious (I), recovered or dead (R) (i.e., a SIIR model). The model splits the patients who have become symptomatic [30, 31] into three categories of isolation: hospitalization, effective home isolation (including safe burials), and no effective isolation (the main assumptions in the model are discussed in the Additional file 2: Appendix S1).

We selected 22 cities from countries with the highest air traffic volume from Liberia, Sierra Leone, and Guinea [12] between September and December 2013 and 2014 (Additional file 2: Appendix S3, Table S8 and Figure S3), excluding high-income countries. These cities were categorized using the World Bank classification of the world’s economies based on per capita gross national income (GNI) for the previous year [32]: Low (< USD 1045), Lower-Middle (USD 1045–USD 4124), Upper-Middle (USD 4125–12735) and High (USD 12736+). As a reference, the GNI per capita of Liberia, Sierra Leone, and Guinea in 2013 was USD 370, USD 730, and USD 450, respectively [32] (Additional file 2: Appendix S2, Table S3).

We estimated low and high case scenarios using a combination of hypothesized initial (seeding) cases that would occur before detection and initiation of an effective response (low seed: 10 cases; high seed: 100 cases), and three rate-of-outbreak growth scenarios (Additional file 2: Appendix S2). We based the seed numbers on two factors. First, observations from West Africa that one or two initial cases can result, due to local customs related treatment of sick persons and burial ceremonies, in several additional cases. As an example of an extreme situation, a single undiagnosed (missed) Ebola case and unsafe burial in Kono, Sierra Leone, resulted in 43 confirmed cases [33]. Further, the degree of underreporting in West Africa was estimated to be between approximately 1.5 to 3.0 Ebola cases for each reported case [34, 35]. Thus, even with 2 identified cases there are likely to be many more initially unidentified cases that represent a risk of onward transmission.

There are multiple factors that may affect both how quickly an Ebola case or outbreak would be detected and how fast an effective response can be initiated. These include, for example, the quality of the disease surveillance system, availability of trained healthcare workers, laboratory and diagnostic capabilities, health infrastructure, accessibility to infected patients, patients’ health-seeking behaviour, available resources, competing government priorities [36–40]. We used three rate-of-growth scenarios to model how quickly an outbreak is contained; these scenarios were based on transmission-and-containment patterns observed in Liberia during the 2014–2016 Ebola epidemic [2] (Additional file 2: Appendix S2). For all three scenarios, we assumed that within the first week of outbreak detection 10% of Ebola cases would be hospitalized or effectively isolated [2, 10] (Additional file 2: Appendix S2, Figure S2). We based the “Liberia-like scenario” on data collected in Liberia during the 2014–2015 Ebola epidemic (3). We assumed a 5–6% increase per week in the number of cases hospitalized or effectively isolated during weeks one through 11, and a 2% increase per week during weeks 12 through 16. This resulted in 66% of cases being effectively isolated by week 15 (3). It is important to note that the Ebola outbreak response effort in Liberia included substantial support from international health workers and organizations, in addition to local response capabilities [2, 3, 16]. We built the “delayed-response” (slower than Liberia) scenario by assuming a 1.5% increase per week in the number of cases hospitalized or effectively isolated during weeks one through three, and 2–4% increase in hospitalization or effective isolation during weeks four through 16. For our “fast-response” scenario, we assumed a 10% increase per week in the number of cases hospitalized or effectively isolated during weeks one through four, a 7% increase per week during weeks five through seven, and a 4% increase per week in weeks eight through 12. In this “fast response” scenario, the final proportion of cases in effective isolation leveled off at 81% at week 13 (Additional file 2: Appendix S2, Figure S2). We used data from Liberia because in August–September 2014, Liberia experienced rapid growth in cases (doubling of cases approximately every 23 days) [41], clearly illustrating some of the challenges faced by countries with limited public health infrastructure and existing capacity for rapid response. Model parameters can be adjusted by the user; the spreadsheet-based model is available for free at http://stacks.cdc.gov/view/cdc/24900.

Given the correlation between GNI per capita and health expenditures [42–44], we assumed that each country’s World Bank economic category was directly related to the ability of that country to respond to an Ebola outbreak. That is, we assumed that higher-income countries would be able to mobilize resources more quickly than Liberia, and lower-income countries would respond more slowly (i.e., we used a three-tiered approach: delayed response, Liberia-like response, and fast response). Higher income countries tend to have a more robust public health infrastructure, laboratory and diagnostic capabilities, and larger trained workforce per capita than lower income countries, which would presumably allow them to implement effective outbreak control measures faster [42–46]. We also assumed that the spread of Ebola was similar among different cities in the same economic category, as they were likely to have comparable health expenditures and infrastructure.

The risk of Ebola transmission between humans is affected by several factors, including the strength of public health and health care systems, behavioral factors such as burial practices, human mobility, and social cohesion, and sociodemographic factors such as population density, substandard housing, and lack of sanitation [38–40, 47–50]. For example, in an analysis of data from Montserrado County, Liberia, cases in areas with lower socio-economic status (SES) were responsible for higher numbers of reported contacts, secondary cases and wider spread to other parts of the county [51]. To account for these differences in living conditions between Monrovia, Liberia, and the cities used in the analyses, we performed two additional sets of analyses. We weighted the estimated number of Ebola cases that would occur using the ratio of: 1) each city’s population density (pop/sq. mile) to Monrovia’s population density (more densely populated cities would have on average more Ebola transmission), and 2) the ratio of the proportion of the population living in slums (shanty towns) at the country level to the proportion of the population living in slums in Liberia.

Last, we selected five countries for modeling the potential of spread of Ebola within a country once a case had been imported into the main city: Nigeria, Ethiopia, Kenya, South Africa and India. These countries were selected as an illustration, based on total population, air travel, population in slums, and with different levels of income. Within each country, we selected major urban centers based on population size (100,000 people or more) and travel access to the country’s major urban area through air travel or location along a major highway. We projected the number of cases in each city, using the same intervention scenarios as before: delayed response for lower income countries, Liberia-like response for lower-middle income countries, and fast response for upper-middle income countries. Despite Liberia being a low-income country, the response to the Ebola outbreak received substantial external support [52]. To account for differences in living conditions, we weighted the estimated number of Ebola cases that could have occurred adjusting by the city’s population density (pop/sq. mile) compared to Monrovia.

**Fig. 1:**
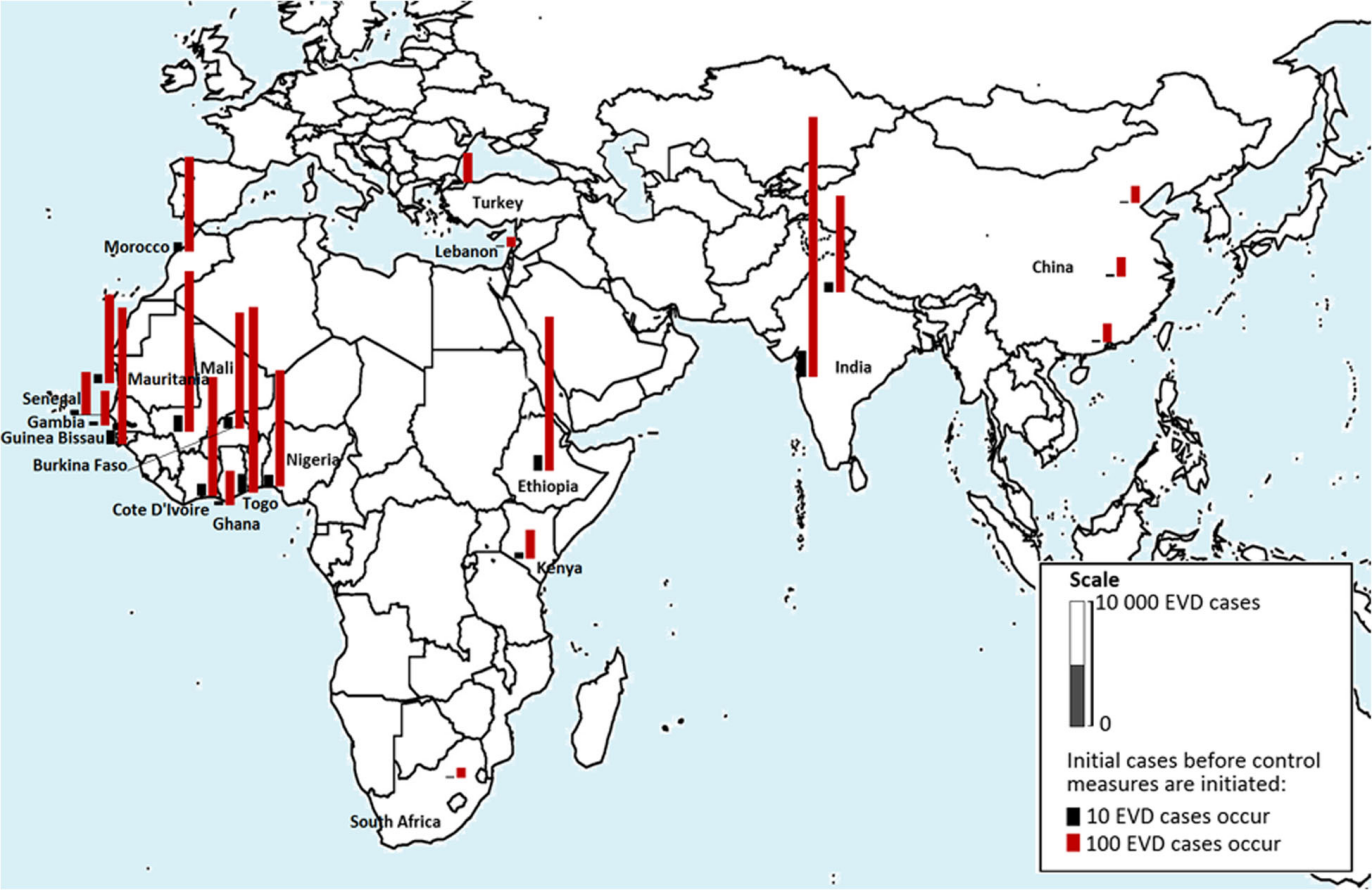
Illustrative scenarios showing the potential broad-scale transmission of Ebola to cities in countries beyond Sierra Leone, Guinea, and Liberia. *Notes: Scenarios showing potential number of cases in each city assume either 10 or 100 cases before detection and initiation of an effective response. Further, in each city, the speed at which an Ebola outbreak was assumed to be brought under control was modeled using one of three control scenarios. These scenarios were either faster, equal or slower than the speed of the control of the Liberian Ebola epidemic, and the estimates adjusted for population density. Cities were allocated to each control scenario based on their country’s World Bank economic classification (see Additional file* *2* *for further details). The complete results of the estimated number of cases in each country for all control scenarios can found in the Additional file* *2* *: Appendix S2, Table S6* [32]

Figure 1 shows the potential number of Ebola cases in each country’s major city, assuming a single case was imported from one of the three Ebola-affected countries, and new ongoing transmission was established. The data mapped in Fig. 1 represent: (i) a delayed-response scenario for low-income countries (Ethiopia, Gambia, Guinea-Bissau, Burkina Faso, Mali, Togo); (ii) a Liberia-like response scenario for lower middle-income countries (Kenya, Mauritania, Senegal, India, Cote D’Ivoire, Ghana, Nigeria, Morocco); and (iii) a fast response scenario for upper-middle income countries (Turkey, China, South Africa, and Lebanon), adjusted by the city’s population density compared to Monrovia. In each country, the black bars represent the estimated number of cases if effective control measures (including having patients receive specialized care in hospitals, effectively isolating people at risk at home or in their communities, safe burial practices, contact tracing and monitoring), were initiated after 10 cases had occurred while the red bars represent what might have happened if the initiation of effective control measures did not occur before there were 100 Ebola cases within the city. For comparison, on March 2014, the WHO announced an Ebola outbreak notified by the Ministry of Health of Guinea, and Liberia reported the first Ebola cases about a week later. At the end of May, presumably knowing that Ebola was a major threat from neighboring Guinea and Liberia, Sierra Leone reported the first Ebola cases, from participants in a local funeral. Five weeks later, by July 11, there were more than 300 confirmed cases of Ebola in Sierra Leone [2]. Further details showing the unadjusted number of Ebola cases, estimated cases weighted by population density, and estimated cases weighted by population living in slums, compared to Liberia are show in the Additional file 2: Appendix S2, Table S6. The main conclusions from the analysis did not change based on the use of weights.

**Fig. 2:**
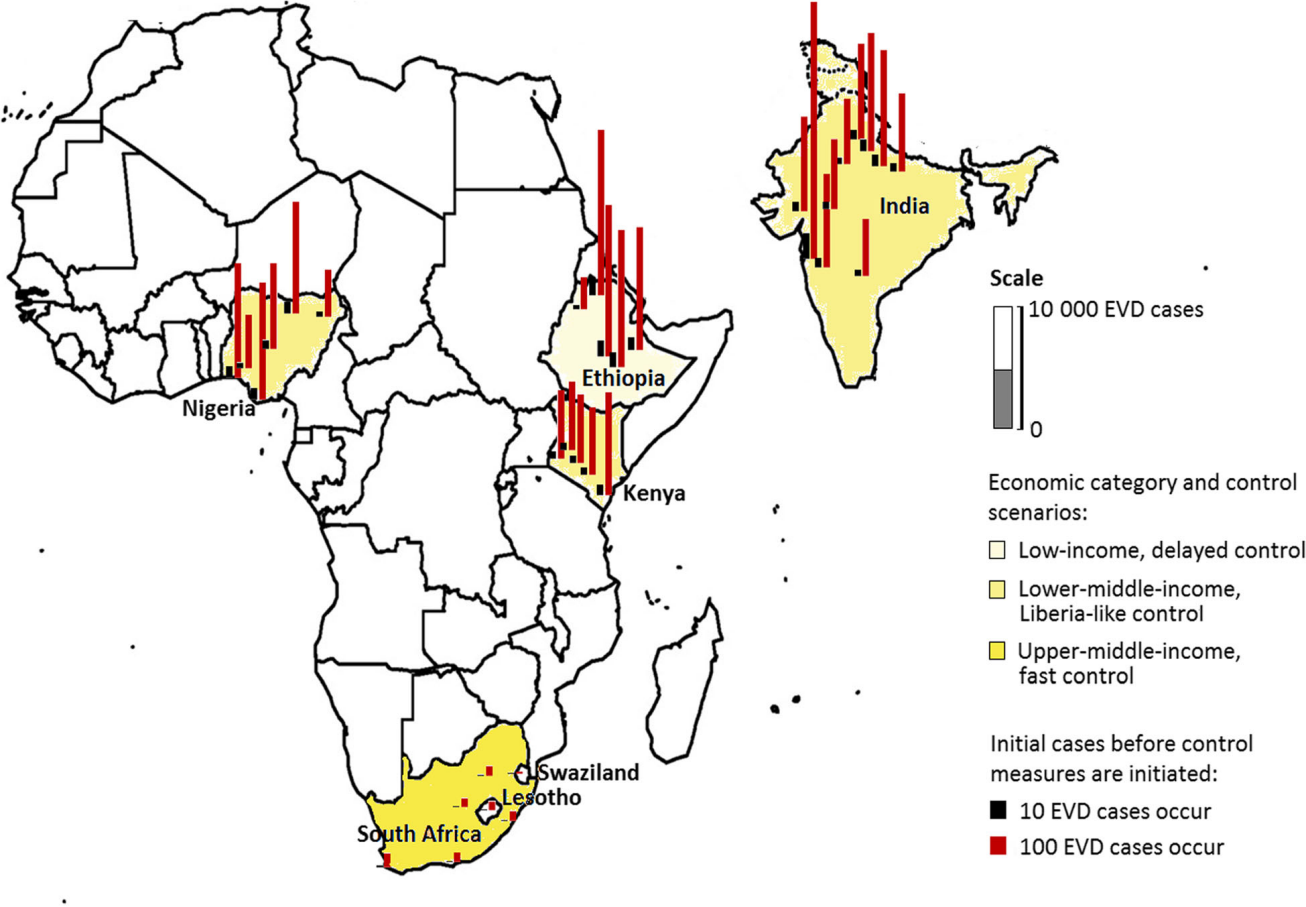
Ebola cases per city in case of intra-country transmission (selected countries). *Note: Scenarios showing potential number of cases in each city assume either 10 or 100 cases before detection and initiation of an effective response. In each city, the speed at which an Ebola outbreak was assumed to be brought under control was modeled using one of three control scenarios. These scenarios were either faster, equal or slower than the speed of the control of the Liberian Ebola epidemic, and the estimates adjusted for population density. Cities were allocated to each control scenario based on their country’s World Bank economic classification (see text and Supplemental material for further details). The specific numbers in this figure are shown in Additional file* *2*: *Appendix S2, Table S7*

Figure 2 shows estimates for the potential number of Ebola cases per city, had Ebola transmission spread from the initial city affected to other major cities within the selected countries (Nigeria, Ethiopia, Kenya, South Africa and India), weighted by population density. Considering the model assumptions, early detection of cases and rapid initiation of control measures would be particularly important in the major cities of low-income countries (Ethiopia). The specific estimates of potential Ebola cases per city and the estimates with no adjustments are shown in the Additional file 2: Appendix S2, Table S7. Our estimates assume that international and/or national preparedness and response efforts were not already substantially underway; i.e., that some number of Ebola cases would occur in a country before the outbreak was detected and an effective response initiated.

### Discussion: Lessons from the potential broad-scale transmission of Ebola

We provide estimates of the number of Ebola cases that could have occurred had the Ebola crisis spread beyond the West African foci. Our estimates illustrate the importance of building early detection and effective response capabilities. Had Ebola spread beyond Sierra Leone, Guinea, and Liberia, the number of cases worldwide could have been several folds higher than observed. The potential spread of Ebola varied greatly, largely hinging on the number of cases that occurred before detection and initiation of an effective response, and on the effectiveness of the response. Preparing for the next crisis entails examining the factors that allowed halting the outbreak in the countries where importations occurred, and analysing the main challenges in the three West African countries where Ebola spread.

A useful illustration of how local health systems can quickly detect and effectively respond to a rapidly evolving outbreak was the control of Ebola in Nigeria in July 2014 [3, 15, 18]. A cluster of 19 Ebola cases originated from a single traveler with Ebola who flew on a commercial airplane from Liberia to Lagos. One Ebola-infected nurse who cared for the index case apparently travelled to Enugu, over 500 km from Lagos, and one primary contact of the index case with Ebola travelled to Port Hartcourt, over 600 km from Lagos [15]. Both cases underscored the risk of rapid intra-country spread of Ebola. The Nigerian government, in collaboration with CDC and other partners, rapidly created an incident management system largely using staff from the Nigerian Polio Eradication Program and support from the Bill and Melinda Gates Foundation. An Emergency Operation Center (EOC) had been recently established in Nigeria to support the polio eradication initiatives, which had prioritized health system strengthening and emergency response preparedness. The polio EOC deputy became the Ebola EOC incident manager, and had readily access to trained staff and financial resources within the health system and from partner agencies including WHO, CDC, and Doctors Without Borders [18]. The response team identified 898 contacts who were followed-up due to potential exposure to Ebola virus; patients with suspected infection were effectively isolated in Ebola treatment facilities [15, 18]. Contact tracing efforts were supervised by experienced epidemiologists and supported by in-country laboratory testing (reverse transcription-polymerase chain reaction and anti-Ebola virus immunoglobulin G diagnostic tests). This rapid initiation of control measures (which included training of healthcare workers, contact tracing, household visits, effective isolation of infectious patients, airport screening, and the creation of an Emergency Treatment Unit in two weeks) was critical to halt Ebola virus transmission, and probably prevent thousands of additional Ebola cases [15]. Our model (Additional file 2: Appendix S2, Table S3) suggests that we would have expected 367 cases of Ebola, if only 10 cases of Ebola had occurred in Nigeria before detection and initiation of an effective response, based on a “Liberia-like” Ebola transmission and response. However, partly due to the enhanced response capabilities from the Nigerian Polio Eradication Program, only one Ebola case occurred before detection and effective response, and Nigeria had a fast response due to availability of resources and trained health workforce. Using these values, to describe a unique scenario, our model predicted 14 Ebola cases (36 using population weights), which is close with what occurred in Nigeria (19 cases of Ebola after a quick and effective response). Our estimates are also comparable to the results from a model based on the days before the intervention, assuming 12 exposed individuals from an index case [15].

Conversely, the outbreak gained ground in Guinea, Liberia, and Sierra Leone due to at least four conditions: slow Ebola detection given lack of Ebola-specific knowledge in the region, initial dearth of preventive and treatment options available, limited national public health systems – despite the countries’ formal commitment to IHR – and the lack of an effective, organized response. The limitations of the local and global response further resulted in the rapid increase of Ebola case counts and deaths, and the inability to respond to other local health care concerns such as malaria, HIV, childhood vaccination, and maternity services [2, 4, 53, 54].

Our model and previous estimates [10] suggest that the Ebola outbreak in West Africa could have potentially been much larger, had local and international health workers and organizations not committed to a major response effort. Our illustrative estimates are subject to at least three limitations. First, extrapolating disease transmission in Liberia to other countries with very different public health systems may not be appropriate, as other factors such as public awareness and attitudes, or spontaneous behavioral changes could alter epidemic growth patterns. Second, we relied on constant epidemiological parameters (e.g., incubation period) for the Ebola outbreak across cities and countries. Third, we used three epidemic growth scenarios and assumed they depended on a country’s GNI, based on an association between GNI per capita and health expenditures [42–44], which may not be reflective of the outbreak detection and response capabilities of some countries. There is substantial uncertainty in our estimates; we have no data to accurately predict the capacity of countries that were at high risk of Ebola transmission, as determined by air travel volume, of detecting and initiating an effective response to prevent or slow down an Ebola outbreak, and there is no counterfactual to contrast our results. However, even though we cannot know whether the estimated cases of Ebola that might have occurred had Ebola spread beyond Sierra Leone, Guinea, and Liberia are accurate estimates, the results underscore the global risk posed by the Ebola crisis and potentially by other infectious disease threats [15, 19, 21–28, 55–59].

The contrast between the Nigerian response and that of the three countries affected by Ebola in West Africa provides a key lesson for future responses: if serious disease outbreaks are detected early, and responses implemented fast and effectively, the risk of a major outbreak requiring an international intervention decreases dramatically. More importantly, thousands of infections and premature deaths are potentially averted. The impact of early detection and response efforts show the importance of strengthening the public health systems of low- and middle- income countries, before infectious disease threats occur [9, 14, 52, 60–62].

The strengthening of public health systems includes: the addition of health personnel; bolstering primary and critical healthcare facilities and public health infrastructure, such as laboratories; and improvements in disease surveillance systems and data collection for situational awareness (for example, number of cases, severity, location, treatment setting) [9, 62, 63]. Preparation also comprises consolidating and assembling a cadre of rapidly deployable responders, and providing them with training and simulation exercises for adequate and timely response (Table 1). With strengthened health systems, and thus capabilities for a timely detection of a public health threat, infectious disease outbreaks would still occur, but their rapid escalation becomes much less likely, and outbreaks could more readily be contained.

**Table 1:** Characteristics of the response to the 2014–2016 Ebola outbreak and actionable lessons learned for local and global response preparedness and capabilities^a^

Characteristics of the 2014–2016 Ebola Outbreak that increased the risk of spread beyond West Africa	Challenges and suggested measures for local and global response preparedness
Slow detection of Ebola	**Local:** Strengthen disease surveillance systems for a quick detection of an infectious disease outbreak. Focus surveillance efforts on specific diseases. Define the type of incentives and capabilities needed to facilitate rapid and transparent reporting of surveillance data. **Global:** Support initial stages of training for surveillance, detection, and response and building surveillance capabilities in low- and middle-income countries.
Lack of a coherent, predictable, organized global response	**Local:** Build capabilities for diagnostics, situational awareness data (cases, severity, treatment setting), and data analysis and reporting. Prepare a cadre of “basic responders”, i.e., community-level health workers, with essential basic training to assist early detection and response efforts in communities, including education and awareness. **Global:** Define the minimum data needed to track disease incidence, spread, and the effects of intervention. Support initial training of “basic responders”. Support emergency response training and simulation exercises for preparedness at local and global level. Define who will coordinate response efforts and make decisions if global response was necessary. Define the type of IT resources are needed to coordinate a response (e.g., electronic connectivity, telecommunications).
Limited national public health systems and, closely related, potential inability of the international community to respond to multiple outbreak foci, beyond West Africa	**Local:** Strengthen public health systems of low and middle- income countries, including health personnel, primary and critical healthcare facilities, and laboratories. Establish procedures and personnel to staff and operate Emergency Operation Centers. **Global:** Support training of public health workers, development of infrastructure and capabilities, and assessment of public health interventions. Train and maintain rapid response teams, including health workers, epidemiologists, managers, ready for quick deployment in case of a threats of international concern, including natural disasters. Secure infrastructure for the quick deployment of Emergency Operation Centers in case of a threats of international concern. Coordinate intercountry and interagency efforts to achieve maximal coverage. Define and secure funding for response workers’ insurance, compensations, and potential evacuation costs.
Inability to concurrently support multiple public health programs in West Africa, including malaria, HIV, childhood vaccination, and maternity services.	**Local:** Strengthen health systems including health personnel, primary and critical healthcare facilities. **Global:** Define emergency migratory policies and strategies that need to be strengthened/modified/adapted to respond to biological threats.
Insufficient preventive and treatment technologies available	**Global:** Strengthen research on emerging infectious diseases and public health threats, above and beyond market forces, including preventive technologies, diagnostics, and protective equipment for health workers. Establish guidelines, preparedness and training for diseases with insufficient treatment, including quarantine and isolation, contract tracing, safe burials, community awareness. Define infrastructure and specific resources requirements needed to implement alternative to treatments (e.g., Ebola Treatment Units).
Larger outbreaks need international, external funding	**Global:** Ensure a reliable intercountry and interagency response. Define and secure sustainable sources of funding.

^a^Table was informed by the Ebola response efforts and previous research and reports, including [3, 9, 14, 52, 60–62]

The recently created Global Health Security Agenda (GHSA) seeks to improve country capacity to prevent, detect, and effectively respond to major public health threats by focusing on strengthening four major components of the public health system: disease surveillance systems, diagnostic capacity, healthcare workforce development, and the establishment of EOCs, all of which proved to be critical in responding to the Ebola crisis. Infectious diseases can spread rapidly, as shown by the Ebola epidemic, H1N1 influenza [56, 57], severe acute respiratory syndrome (SARS) [58], and, more recently, by the regional spread of the Zika virus [59].

## Conclusions

Overall, the Ebola outbreak could have potentially been much larger had local and international health workers and organizations not committed to a major response effort. The outbreak proved that global health security needs to be a priority, and confirmed, once again, that increased mobility, air travel, and international trade have increased our connections as a global community and that we share the health risks of emerging and re-emerging pathogens and other public health threats [52]. The great paradox is that while the world may now be better prepared than ever in history to respond to emerging threats, we are at a higher risk of pandemics from increasing interconnectivity [3]. Health security has to be founded on robust local health systems that can rapidly detect and effectively respond to an infectious disease outbreak. When local capacity is insufficient, an effective global health response has to be quick and decisive, with all parties working cooperatively and in a coordinate fashion [64]. While the Ebola epidemic was unprecedented in many ways, the lack of a coherent, organized, and timely response, highlighted critical questions that need an urgent response.

## Additional files

Additional file 1: Multilingual abstracts in the six official working languages of the United Nations. (PDF 490 kb)

Additional file 2: Technical appendix and supplemental material for the potential broad-scale transmission of Ebola virus disease during the West Africa crisis. (PDF 10027 kb)

## Abbreviations

CDC: Centers for Disease Control and Prevention
EOC: Emergency Operation Center
GDP: Gross Domestic Product
GHSA: Global Health Security Agenda
GNI: Gross National Income
HIV: Human Immunodeficiency Virus
IHR: International Health Regulations
SARS: Severe Acute Respiratory Syndrome
WHO: World Health Organization
