# Supplementary material for: Potential for broad-scale transmission of Ebola virus disease during the West Africa crisis: lessons for the Global Health security agenda
Source: Infect Dis Poverty. 2017 Dec 1;6:159. doi: 10.1186/s40249-017-0373-4 (PMC5710062; doi:10.1186/s40249-017-0373-4)
Supplement: Supplementary file 1 — Multilingual abstracts in the six official working languages of the United Nations. (PDF 490 kb) [file 40249_2017_373_MOESM1_ESM.pdf]

Translation of the abstract into the six official working languages of the United Nations

إمكانية انتقال مرض فيروس إيبولا على نطاق واسع خلال أزمة غرب أفريقيا: دروس لبرنامج الأمن الصحي العالمي

إدواردو أوندوراجا، كريستينا كارياس، مارتن إ. ملتزر، وإميلي ب. كاهن

ملخص
خلفية: أدت أزمة الإيبولا في الفترة 2014-2016 في غرب أفريقيا إلى ما يقرب من ثمانية أضعاف عدد الوفيات المبلغ عنها باعتبارها مجموع جميع حالات تفشي الإيبولا السابقة. انتشار وباء حالات الإيبولا المتعددة في سبع دول على الأقل خارج ليبريا، وسيراليون، وغينيا، إلى احتمال انتقال فيروس إيبولا على نطاق واسع.
النص الرئيسي: وباستخدام أداة النمذجة التي وضعتها مراكز الولايات المتحدة لمكافحة الأمراض والوقاية منها خلال تفشي فيروس إيبولا، قدرنا عدد حالات الإيبولا التي كان يمكن أن تحدث لو أن انتشار المرض خارج الدول الثلاث في غرب أفريقيا إلى مدن في دول أخرى معرضة لخطر انتقال المرض (استنادا إلى أنماط السفر الجوي نهاية عام 2014). لقد قدرنا حالات الإيبولا في ثلاثة سيناريوهات: استجابة متأخرة، واستجابة شبيهة بليبريا، وسيناريو استجابة سريعة. واستنادا إلى تقديراتنا لعدد حالات الإيبولا التي كان يمكن أن تحدث لو ان انتشار فيروس إيبولا وصول لدول أخرى خارج بؤر غرب أفريقيا، نؤكد على الحاجة إلى تحسين مستويات التأهب والاستجابة لتهديدات الصحة العامة، وهو هدف الأجندة العالمية للأمن الصحي. وتشير نتائجنا إلى أن الإيبولا يمكن أن ينتشر على نطاق واسع خارج بؤر غرب أفريقيا، في حالة عدم التزام العاملين الصحيين المحليين والدوليين والمنظمات ببذل جهود استجابة عالية. وتؤكد نتائجنا أهمية الكشف السريع والاستجابة الفعالة والمنظمة والتحديات التي تواجهها الدول ذات النظم الصحية العامة المحدودة. وتشمل الدروس العملية لتعزيز نظم الصحة العامة المحلية في الدول المعرضة لخطر انتقال الأمراض زيادة العاملين الصحيين وتعزيز مرافق الرعاية الصحية الأولية والحرجة وتطوير الهياكل الأساسية للصحة العمومية (مثل القدرة المعملية) وتحسين مراقبة الأمراض. ومع وجود نظم محلية أقوى للصحة العامة، فإن تفشي الأمراض المعدية سيظل قائما، ولكن تفشيها السريع سيكون أقل احتمالا إلى حد كبير، مما يقلل من أثر تهديدات الصحة العامة مثل فيروس إيبولا.
الاستنتاجات: من المحتمل أن ينتشر تفشي فيروس إيبولا إلى دول أخرى، حيث قد تكون قدرات المراقبة والاستجابة الصحية العامة المحدودة قد أدت إلى لوجود بؤر إضافية. ويتطلب الأمن الصحي وجود نظم صحية محلية قوية يمكنها الكشف بسرعة عن تفشي الأمراض المعدية والاستجابة لها بفعالية.

Translated from English version into Arabic by Mahmoud Sami, through

TRANSLATORS
WITHOUT BORDERS

在西非埃博拉暴发期间大规模传播埃博拉病毒疾病的可能性：全球卫生安全议程的教训

Eduardo A. Undurraga, Cristina Carias, Martin I. Meltzer, Emily B. Kahn

摘要
引言：2014 - 2016 年西非的埃博拉病毒病疫情报告的死亡病例数是以前的所有埃博拉病毒病暴发总和的 8 倍。在利比里亚、塞拉利昂和几内亚以外的至少 7 个国家，暴发事件和多起埃博拉病毒病例的发生均提示埃博拉病毒大范围传播的可能性。

主要内容：使用美国疾病预防控制中心在埃博拉暴发期间研发的建模工具，根据 2014 年底的航空旅行模式，我们估算了可能出现的埃博拉病例数，该病蔓延到西非 3 个国家以外的其他国家的高传播风险城市。我们估计埃博拉病例疫情响应有 3 种情况：延迟响应、类似利比里亚的反应和快速反应。根据我们对埃博拉病毒蔓延到西非疫情暴发点以外的其他国家可能发生的埃博拉病例数量的估计，我们强调需要加强对公共卫生威胁的准备和应对措施，这是全球卫生安全议程的目标。结果表明，埃博拉病毒可能潜在地扩散到西非疫情暴发点之外，本地和国际卫生工作者和组织未能积极应对。本研究还强调了快速检测和启动有效、有组织的应对措施的重要性，以及公共卫生体系不完善国家所面临的挑战。在疾病传播高风险国家应加强当地公共卫生体系建设，包括增加卫生人员，完善初级和关键卫生保健设施，发展公共卫生基础设施（如实验室能力）和提高疾病监测能力。随着地方公共卫生体系的建设，传染病疫情仍将持续发生，但其迅速升级的可能性将会大大降低，可以尽可能减少埃博拉等公共卫生事件的影响。
结论：埃博拉疫点可能会扩散到其他公共卫生监测和应急能力有限的国家，可能出现更多的疫情暴发点。公共卫生安全需要强大的当地卫生体系支撑，可以快速检测并有效应对传染病疫情。

Translated from English version into Chinese by Lei Sun, edited by Pin Yang

CDC CHINESE CENTER FOR DISEASE CONTROL AND PREVENTION
NATIONAL INSTITUTE OF PARASITIC DISEASES

Potentiel de transmission à grande échelle du virus Ebola pendant la crise en Afrique de l'Ouest : leçons pour le programme de sécurité sanitaire mondiale.

Eduardo A. Undurraga, Cristina Carias, Martin I. Meltzer, Emily B. Kahn

Résumé
Contexte: La crise de l'Ebola 2014-2016 en Afrique de l'Ouest a rapporté environ huit fois plus de décès que la somme de toutes les épidémies précédentes d'Ebola. L'ampleur de l'épidémie et l'occurence de cas multiples d'Ebola dans au moins sept pays au-delà du Libéria, de Sierra Leone et de la Guinée, laisse entrevoir la possibilité d'une transmission à grande échelle d'Ebola.
Texte principal: À l'aide d'un outil de modélisation mis au point par les centres américains de prévention et de contrôle des maladies lors de l'épidémie d'Ebola, nous avons estimé le nombre de cas d'Ebola qui auraient pu se produire si la maladie s'était propagée au-delà des trois pas d'Afrique de l'Ouest vers des villes d'autres pays à haut risque de transmission de la maladie (sur la base des modèles de transport aérien à la fin de 2014). Nous avons estimé les cas d'Ebola selon trois scénarios: une réaction tardive, une réaction semblable à celle du Libéria et un scénario de réaction rapide. Sur la base de nos estimations du nombre de cas d'Ebola qui auraient pu se produire si l'Ebola s'était propagé à d'autres pays au-delà des foyers ouest-africains, nous soulignons la nécessité d'améliorer les niveaux de préparation et de réponse aux menaces pour la santé publique, ce qui est l'objectif du Programme mondial pour la sécurité sanitaire. Nos résultats suggèrent que l'Ebola aurait pu se propager largement au-delà des foyers de l'Afrique de l'Ouest, si les agents de santé locaux et internationaux et les organisations n'étaient pas engagés dans un effort d'intervention majeur. Nos résultats soulignent

l'importance d'une détection rapide et de la mise en place d'une réponse efficace et organisée ainsi que les défis auxquels sont confrontés les pays dont les systèmes de santé publique sont limités. Pour renforcer les systèmes locaux de santé publique dans les pays à haut risque de transmission de maladies, il est possible de tirer des leçons pratiques, notamment en augmentant le personnel de santé, en renforçant les établissements de soins de santé primaires et essentiels, en développant les infrastructures de santé publique (ex : la capacité des laboratoires) et l'amélioration de la surveillance des maladies. Avec le renforcement des systèmes locaux de santé publique, les épidémies de maladies infectueuses se produiraient encore, mais leur propagation rapide serait beaucoup moins probable, ce qui minimiserait l'impact des menaces pour la santé publique comme dans le cas de l'Ebola.

Conclusions: L'éclosion d'Ebola aurait pu se propager à d'autres pas, où les capacités limitées de surveillance et d'intervention en matière de santé publique auraient pu entraîner des foyers supplémentaires. La sécurité sanitaire exige des systèmes de santé locaux robustes capables de détecter rapidement une éclosion de maladies infectueuses et d'y réagir efficacement.

Translated from English version into French by Johanne Jean-Maître, through

TRANSLATORS
WITHOUT BORDERS

Потенциал широкомасштабной передачи вируса Эболы в период кризиса в Западной Африке: извлечённые уроки в рамках Программы по обебспечению безопасности в области глобального здравоохранения

Эдуардо А. Ундуррада, Кристина Кариас, Мартин И. Мельцер, Эмили Б. Кан

Аннотация

Справочная информация: В период кризиса Эболы в 2014-2016 годах в Западной Африке было зарегистрировано примерно в восемь раз больше случаев смерти нежели сумма всех предыдущих вспышек заболевания. Масштабы вспышек, а также появление множественных случаев Эболы по меньшей мере в семи странах за пределами Либерии, Сьерра-Леоне и Гвинеи, указали на возможность широкомасштабной передачи заболевания.

Основной текст: Используя инструмент моделирования, разработанный Центрами США по контролю и профилактике заболеваний во время вспышки Эбола, мы подсчитали количество случаев Эбола, которые могли бы произойти, если бы болезнь распространилась за пределы трех стран Западной Африки в города других стран с высоким риском для (основываясь на маршрутах воздушных перевозок конца 2014 года). Мы оценили случаи Эбола в трех сценариях: как запоздалую реакцию, как реакцию, аналогичную Либерии и сценарий быстрого реагирования. Основываясь на оценках потенциального числа случаев Эбола в случае, если бы заболевание распространилось на другие страны за пределами западноафриканских очагов, мы подчеркиваем необходимость повышения уровня готовности и реагирования на угрозы

общественному здравоохранению, что является целью Программы по обеспечению безопасности в области глобального здравоохранения. Наши результаты показывают, что вирус Эбола потенциально мог распространиться далеко за пределы очагов Западной Африки, если бы местные и международные медицинские работники и организации не приложили бы значительные усилия по оперативному реагированию. Наши результаты подчеркивают важность оперативного выявления и инициирования эффективного, организованного реагирования, а также решения проблем, с которыми сталкиваются страны с ограниченными системами общественного здравоохранения. Эффективные уроки для укрепления местных систем общественного здравоохранения в странах с высоким риском передачи болезней включают в себя увеличение штата медицинского персонала, укрепление первичных и критических медицинских учреждений, развитие инфраструктуры общественного здравоохранения (например, лабораторного потенциала), а также совершенствование эпиднадзора за болезнями. При наличии более сильных местных систем общественного здравоохранения вспышки инфекционных заболеваний хотя и будут возникать, но их быстрое распространение будет значительно менее вероятным, сведя к минимуму угрозу общественному здравоохранению от таких заболеваний, как Эбола.

Выводы: Вспышка Эболы потенциально могла распространиться на другие страны, где ограниченные возможности наблюдения и реагирования на общественное здравоохранение могли привести к дополнительным очагам заражения. Безопасность здравоохранения требует надежных местных систем здравоохранения, которые в состоянии быстро обнаруживать и эффективно реагировать на вспышки инфекционных заболеваний.

Translated from English version into Russian by Liudmila Tomanek (nee Volynets), through

Potencial de transmisión a gran escala de la enfermedad del virus del Ébola durante la crisis de África Occidental: lecciones para la Agenda Mundial de Seguridad Sanitaria

Eduardo A. Undurraga, Cristina Carías, Martin I. Meltzer, Emily B. Kahn

Resumen

Antecedentes: En la crisis de ébola de 2014-2016 ocurrida en África Occidental se reportaron aproximadamente ocho veces más muertes que la suma de todos los brotes de ébola previos. La magnitud del brote y la aparición de múltiples casos de ébola en por lo menos siete países además de Liberia, Sierra Leona y Guinea, sugieren la posibilidad de una transmisión a gran escala del ébola.

Texto principal: Utilizando una herramienta de simulación desarrollada por los Centros para el Control y la Prevención de Enfermedades durante el brote de ébola, calculamos el número de casos de ébola que podrían haberse producido si la enfermedad se hubiera extendido más allá de los tres países

de África Occidental a ciudades de otros países con alto riesgo de transmisión de la enfermedad (basada en patrones de viajes aéreos tomados a finales de 2014). Hemos realizado una estimación de los casos de ébola en tres escenarios: una respuesta tardía, una respuesta tipo Liberia, y un escenario de respuesta rápida. En base a nuestras estimaciones del número de casos de ébola que podrían haber ocurrido si el virus se hubiera extendido a otros países más allá de los focos de África Occidental, enfatizamos la necesidad de mejorar los niveles de preparación y respuesta a las amenazas a la salud pública, que es el objetivo de la Agenda Mundial de Seguridad Sanitaria. Nuestros resultados sugieren que el ébola podría haberse propagado mucho más allá de los focos de África Occidental, y que los trabajadores y organizaciones de salud locales e internacionales no estaban comprometidos con un gran esfuerzo de respuesta. Nuestros resultados subrayan la importancia de la detección rápida y el inicio de una respuesta eficaz y organizada, y los desafíos que enfrentan los países con sistemas de salud pública limitados. Las lecciones prácticas para fortalecer los sistemas locales de salud pública en los países con alto riesgo de transmisión de enfermedades incluyen el aumento del personal de salud, el fortalecimiento de las instalaciones de atención primaria y crítica, el desarrollo de infraestructura de salud pública (por ejemplo, capacidad de laboratorio) y la mejora de la vigilancia de enfermedades. Aún con sistemas de salud pública locales más fuertes se producirían brotes de enfermedades infecciosas, pero una escalada rápida sería considerablemente menos probable, minimizando así el impacto de amenazas para la salud pública como la del ébola.

Conclusiones: El brote de ébola podría haberse propagado potencialmente a otros países donde la vigilancia de salud pública y las capacidades de respuesta limitadas pueden haber dado lugar a focos adicionales. La seguridad sanitaria requiere sistemas de salud locales robustos que puedan detectar rápidamente y responder eficazmente al brote de una enfermedad infecciosa.

Translated from English version into Spanish by Andrea Lorca, through

TRANSLATORS
WITHOUT BORDERS
