# Supplementary material for: Potential for broad-scale transmission of Ebola virus disease during the West Africa crisis: lessons for the Global Health security agenda
Source: Infect Dis Poverty. 2017 Dec 1;6:159. doi: 10.1186/s40249-017-0373-4 (PMC5710062; doi:10.1186/s40249-017-0373-4)
Supplement: Supplementary file 2 — Technical appendix and supplemental material for the potential broad-scale transmission of Ebola virus disease during the West Africa crisis. (PDF 10027 kb) [file 40249_2017_373_MOESM2_ESM.pdf]

Infectious Diseases of Poverty

Additional file 2

Potential broad-scale transmission of Ebola virus disease during the West Africa crisis: lessons for the Global Health Security Agenda

Undurraga, E.A., Carias, C., Meltzer, M.I., & Kahn, E.B.

Table of contents

1 Appendix S1. EbolaResponse tool: methods and assumptions 2
1.1 Model overview 2
1.2 Model characteristics 3
2 Appendix S2. Broad-scale transmission of Ebola: growth scenarios 7
2.1 Growth scenarios based on transmission patterns 7
2.2 Estimating the number of Ebola cases 9
2.3 Potential broad-scale transmission of Ebola cases in selected cities 12
2.3.1 Estimated number of Ebola cases 12
2.3.2 Estimated number of Ebola cases using city-specific weights 14
2.4 Potential broad-scale transmission of Ebola cases to major cities in within five selected countries 17
3 Appendix S3. Travel data 19

1 Appendix S1. EbolaResponse tool: methods and assumptions

1.1 Model overview

EbolaResponse is a spreadsheet-based model to estimate the number of Ebola cases [1]. The model was developed at an early stage of the Ebola response to aid in planning for additional disease-control efforts. The model facilitated decision-making, for example, by estimating that about 70% of persons with Ebola needed to be in medical care facilities, Ebola Treatments Units (ETUs), or in effective isolation in their communities (including safe burial when needed), or quantitatively estimating the additional deaths from a delay in the response. The model tracks patients through the following states: susceptible to disease (S), infected people incubating Ebola virus (I), infectious (I), recovered or dead (R), i.e., a SIIR model. The model is in effect, a Markov Chain model, and is similar in concept to that built by Chowell et al. [2], but does not include a state for "exposure."

EbolaResponse uses probabilities, drawn from reports of previous Ebola outbreaks [3, 4], to model the daily movement of patients between and within the various defined states. For example, we adapted data from these reports to estimate the duration of the incubation period for Ebola cases, which indicate the probability (likelihood) that patients will incubate one, two, three, or more days, up to a maximum of 25 days (Figure S1).

Figure S1. Distribution of Ebola virus incubation period (from exposure to symptoms), by days of incubation

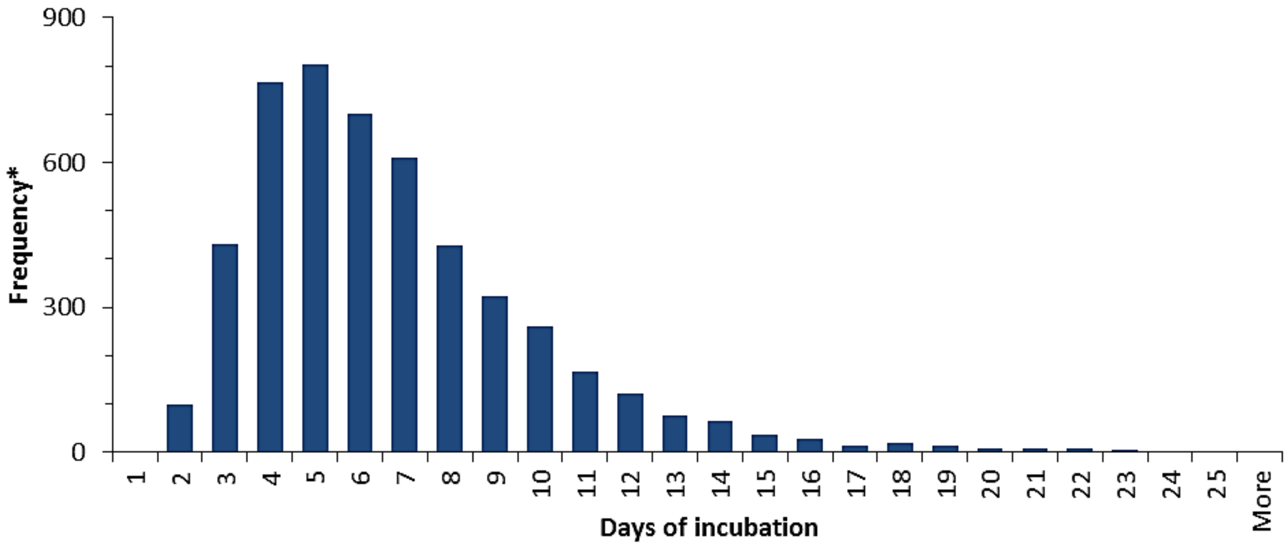

Notes: Adapted from Legrand et al.[3] and Eichner et al. [4]. *Frequency related to the number of patients out of a total of 5,000. **Source:** Meltzer et al.[1]

1.2 Model characteristics

Progression only: EbolaResponse uses specific features to derive the number of Ebola virus disease (EVD) cases. A patient can only progress forward through the model (e.g., can never go from incubating (I) back to susceptible (S)), and no patient can skip a state (e.g., go from incubating to recovered, skipping infectious).

Community size: the model uses a community size equivalent to the total population in the city, and assumes that all the population is susceptible to the disease.

Incubation period: we adapted the *probability distribution* data from Legrand et al.[3] and Eichner et al.[4] to generate a lognormal probability distribution of Ebola incubation (Figure S1). The model uses a mean incubation period of 6.3 days, a median of 5.5 days, and a standard deviation (SD) of 3.31 days.

Chowell et al. [2] estimated mean incubation periods of 5.30 (SD 0.23) and 3.35 (SD 0.49) days based on data from EVD outbreaks in the Democratic Republic of the Congo (formerly Zaire) in 1995

and in Uganda in 2000. These estimated incubation periods are lower than other estimates, such as Legrand et al.[3] and Eichner et al. [4]. These differences may be partly attributed to different virus sub-types [4]. WHO estimated a mean incubation period for the first 9 months of the West African outbreak of 11.4 days, with an upper limit of 21 days [5] and, more recently, Chowell estimated mean incubation period of 12.7 days and mean infectious period of 6.5 days [6]. EbolaResponse allows adjusting the probability distribution to almost any structure desired, with an upper limit of 25 days incubation. While the specific probability distribution used for incubation periods affected the point estimates of the results, they do not affect the overall conclusions of the report.

Infectious period: Several studies of EVD outbreaks have estimated the infectious period to range between 6 to days approximately. The WHO Ebola Response Team estimated the interval from symptoms onset to death was 7.5 days by September 2014 [5] and 8.2 days by the end of November 2014 [7]. A detailed study of chains of transmission between February and August 2014, estimated an infectious period of 8.9 days [8]. A recent parameter review of all EVD epidemics found that the mean time from symptom onset to death in all previous epidemics ranged from 6 to 10.1 days [9], and a modeling study for previous outbreaks estimated 6.5 days as the infectious period [10]. Because the purpose of our estimate is to illustrate what could have happened had the Ebola outbreak of West Africa expanded beyond the three mostly affected countries, we used the best estimate from WHO for the West Africa outbreak, i.e., an infectious period of 8 days [7]. We found no data reporting measurement of changes in the risk of onward transmission over the duration of fulminate illness. It would be conceivable, therefore, that such risk does change as a patient becomes sicker and requires more and more care. We assumed that the risk of onward transmission (infection) from patient to susceptible was equal throughout the 8-day period. Last, burial practices in the region show potential risk of EVD transmission during a traditional burial due to possible contact with a victim's body fluids [11]. Safe

burial practices were considered as a component of the safe community isolation intervention in our model (see section on distribution of patient by category over time).

Population governor: EbolaResponse includes a population "governor" that prevents the model from calculating more cases than the inputted population. This overestimate could happen if one assumes that most of the patients remain in the "not effective home isolation," which has the highest risk of onward disease transmission (Table S1).

The population governor was programmed by simply reducing the daily estimate of the persons newly infected proportionate to the cumulative reduction in the susceptible population, as follows:

Factor to reduce estimate of newly infected at day *t* = [Model population – cumulative total of newly infected up to day (*t*-1)] / model population.

This governor reduces the effective number of persons infected daily (i.e., effectively lowers the risk of transmission inputs shown in Table S1). With "large populations," this governor is unlikely to affect the calculations. The "governor" only begins to appreciably affect estimates (i.e., reduce them) when approximately 40% - 50% of the population have become infected.

Distribution of patient by category over time: The model splits the patients who have become symptomatic [12, 13] into three categories of isolation: (1) hospitalized, (2) effective home isolation, and (3) no effective home isolation. These three categories reflect the ability, or risk, to transmit Ebola onward. The distribution of patients into these categories affects the overall progress of the epidemic. The more patients in the categories "hospitalized," and "effective home isolation", the slower the

progress of the epidemic (because these two categories have transmission rates less than 1 person infected per infectious person). It is possible that a proportion of patients in the “effective home isolation” and the “no effective home isolation” scenarios would end up in the hospital; however, we have assumed that they go so late in the progression of disease as to make no notable change in the risk of onward transmission.

Table S1. Calculated risk for onward transmission of Ebola, by patient category. EbolaResponse tool, West Africa, 2004

Patient category	Daily risk for onward transmission			No. infected per infectious person‡	
	Values used to fit data for Liberia and Sierra Leone*	Values from literature†		From literature (95%CI)	Model estimates
		DRC (95% CI)	Uganda (95% CI)		
Hospitalized	0.02	0.1134 (0.00001-0.5842)	0.0017 (0.00-0.918)	0.4 (0.0-2.2)	0.13
				0.01 (0.0-3.5)	
Home or community setting with reduced risk of transmission§	0.06	0.084 (0.06-0.313)	0.5045 (0.0576-0.5391)	0.5 (0.4-1.9)	0.24
				2.6 (0.3-2.8)	
Home with no effective isolation	0.23	1.0932 (0.00001-1.4281)	0.066 (0.00-3.0367)	1.8 (0.0-2.3)	1.8
				0.1 (0.0-3.2)	

Notes: CI denotes confidence interval; DRC denotes Democratic Republic of Congo.
* Values used to obtain a good fit of cases estimated by the EbolaResponse model to the reported cumulative cases in Liberia and Sierra Leone.
† Values adapted from weekly values given by from Ebola outbreaks in 1995 in DRC (formerly Zaire), and in 2000 in Uganda [3].
‡When these values remain at less than one person infected per infectious person, the epidemic eventually ends. The EbolaResponse modelling tool uses the shown values to fit the model to the data, assuming 6 days of infectiousness.
§This patient category refers to patients at home or in a community setting such that there is a reduced risk for disease transmission (including safe burial when needed).
Source: Meltzer et al. [1].

2 Appendix S2. Broad-scale transmission of Ebola: growth scenarios

2.1 Growth scenarios based on transmission patterns

To estimate the potential broad-scale transmission of Ebola, we modeled three growth scenarios based on transmission patterns observed in Liberia during the 2014-15 EVD epidemic (Figure S2) [14]. For all scenarios we assumed that within the first week of case detection, 10% of the EVD cases would be hospitalized or effectively isolated, as was estimated for the EbolaResponse tool based on reported epidemiological data [1, 14]. We used the EbolaResponse tool to estimate the total number of EVD cases in each of the three scenarios using the parameters summarized in Table S2.

1) **Liberia-like scenario**. Was based on the compartmentalization of Ebola cases that was fitted to data collected in Liberia during the 2014-2015 Ebola epidemic. We assumed a 5-6 percentage points increase per week in the number of cases hospitalized or effectively isolated during weeks one through 11, and a two percentage increase per week during weeks 12 through 16. This resulted in a total of 66% of cases being effectively isolated by week 15.

2) **Delayed-response scenario**. Based on the assumption that the implementation of control measures would proceed slower than observed in Liberia and ultimately reach a smaller proportion of the population, we assumed a 1.5 percentage point increase per week in the number of cases hospitalized or effectively isolated during weeks one through three and between 2-4 percentage point increases per week during weeks four through 16. The final proportion of Ebola cases in effective isolation was 50% at the end of week 16.

3) **Fast-response scenario**. Based on the assumption that control measures would be implemented more quickly compared to Liberia and ultimately reached a larger proportion of the population.

We assumed a 10-percentage point increase per week in the number of cases hospitalized or effectively isolated during weeks one through four, seven percentage point increase per week during weeks five through seven, and a four percentage point increase per week in weeks eight through 12. The final proportion of cases in effective isolation leveled off at 81% at week 13.

Figure S2. Scenarios of delayed, Liberia-like, and fast implementation of control measures to prevent EVD spread used to model the projected number of Ebola cases in a potential scenario of global spread.

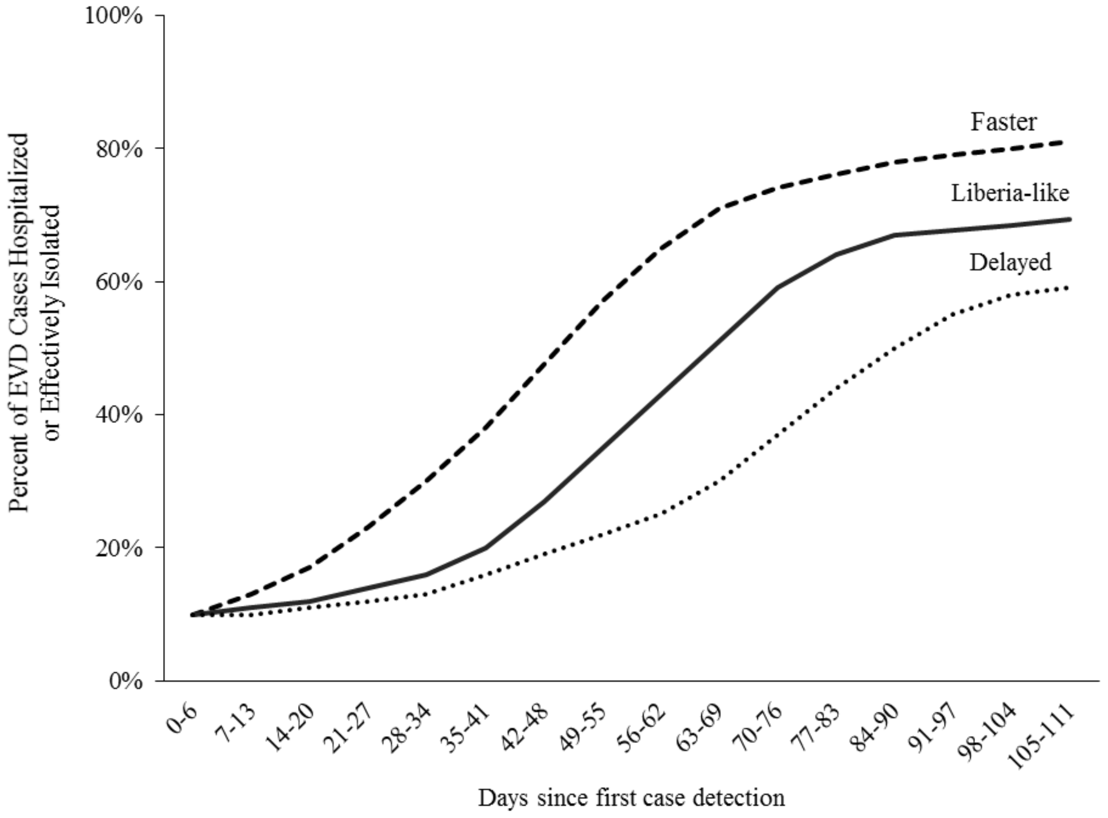

Table S2. Parameters for the three scenarios for Ebola transmission.

Days	Category	Share of patients		
		Delayed	Liberia*	Fast
0-30	Hospitalized	0.06	0.06	0.15
	Effective Home Isolation	0.07	0.10	0.15
	No Effective Home Isolation	0.87	0.84	0.70
	All	1.00	1.00	1.00
31-60	Hospitalized	0.15	0.21	0.20
	Effective Home Isolation	0.10	0.22	0.45
	No Effective Home Isolation	0.75	0.57	0.35
	All	1.00	1.00	1.00
61-90	Hospitalized	0.20	0.30	0.25
	Effective Home Isolation	0.30	0.37	0.53
	No Effective Home Isolation	0.50	0..33	0.22
	All	1.00	1.00	1.00
91-120	Hospitalized	0.25	0.25	0.30
	Effective Home Isolation	0.35	0.45	0.51
	No Effective Home Isolation	0.40	0.30	0.19
	All	1.00	1.00	1.00

Notes: * The estimated implementation of control measures to prevent disease in the baseline Liberia-like scenario was fitted to reported Ebola cases beginning on day 91 of the outbreak, because the data suggests that a large, coordinated response began around this day.

2.2 Estimating the number of Ebola cases

We selected 21 cities with both high volumes of air traffic from West Africa and a high percentage of the city's population living in slums for modeling (Table S3 shows the main characteristics of the cities chosen; air traffic is shown in Appendix 3). To calculate the number of projected cases in each city, we assigned a proportion of patients to either "hospitalized", "effective community isolation" or "no effective isolation" for each of the growth scenarios outlined above. Using the EbolaResponse model [1], we input each city's population and assumed either 10 or 100 cases occur before detection and initiation of an effective response (hereafter seeding). We estimated low and high case count

estimates based on number of cases that occur before detection and initiation of an effective response (hereafter seeding; low case count: 10 cases; high case count: 100 cases). The difference in speed of initiation of an effective response could be due to multiple causes, including the speed of outbreak detection and the speed with which resources can be gathered and deployed. The total number of new cases was then added over 120 days to produce the total cumulative case counts. Bed capacity and diagnostic capabilities are an important limitation to the effectiveness of a response to Ebola [15, 16]. Because it was calibrated with data from the field, the Liberia curve considers these variables as limiting factors in our isolation proportions. The delayed and fast response scenarios thus assume a capacity to expand bed capacity that is slower and faster than Liberia. Because our estimates are only meant to illustrate what could have happened, we are assuming that countries in each category of response could achieve similar capabilities in short time.

Table S3. Characteristics of the cities included in the main analysis

Country	City	City pop. (1,000s)	Pop. density (people/mile^2)*	Economic category of the country†	Healthcare spending‡ (US$/person)	Pop. in urban slums (1,000s) §
Ethiopia	Addis Ababa	3,238	19,900	Low-income	22	467
Gambia	Banjul	504	4,600	Low-income	31	107
Guinea-Bissau	Bissau	492	18,100	Low-income	31	202
Burkina Faso	Ouagadougou	2,741	15,000	Low-income	40	538
Mali	Bamako	2,515	20,800	Low-income	42	571
Togo	Lome	956	24,300	Low-income	48	198
Kenya	Nairobi	3,915	22,000	Lower-middle-inc.	42	567
Mauritania	Nouakchott	968	28,800	Lower-middle-inc.	44	469
Senegal	Dakar	3,520	13,600	Lower-middle-inc.	44	619
India	Mumbai	21,043	83,900	Lower-middle-inc.	58	1,654
India	Delhi	25,703	31,200	Lower-middle-inc.	58	2,021
Cote D'Ivoire	Abidjan	4,860	38,400	Lower-middle-inc.	80	1,491
Ghana	Accra	2,277	11,100	Lower-middle-inc.	86	470
Nigeria	Lagos	13,123	37,500	Lower-middle-inc.	93	3,180
Morocco	Casablanca	3,515	30,600	Lower-middle-inc.	181	279
Turkey	Istanbul	14,164	25,300	Upper-middle-inc.	197	1,243
China	Beijing	20,384	14,200	Upper-middle-inc.	322	2,796
China	Guangzhou	11,265	15,500	Upper-middle-inc.	322	1,545
China	Wuhan	10,220	16,700	Upper-middle-inc.	322	1,402
South Africa	Johannesburg	9,399	8,400	Upper-middle-inc.	651	1,400
Lebanon	Beirut	2,226	8,500	Upper-middle-inc.	663	866
Guinea	Conakry	1,936	28,600	Low-income	22	314
Sierra Leone	Freetown	1,007	14,700	Low-income	22	318
Liberia	Monrovia	1,264	31,100	Low-income	39	429

Notes: * Population density was estimated for each city [17].

† Lower-middle-inc. denotes lower-middle-income country, and upper-middle-inc. denotes upper-middle-income country, as classified by the World Bank [18].

‡Estimates in US$ at average exchange rate in nominal terms (current prices). National Health Accounts are not maintained or updated by all countries; estimates of health expenditures may have been estimated through technical contacts from the country or public documents and reports [19].

§The population living in urban slums is reported at the national level [20]; to derive an estimate for the city we assumed that population living in slums is proportional to population size.

2.3 Potential broad-scale transmission of Ebola cases in selected cities

2.3.1 Estimated number of Ebola cases

Table S4 illustrates the estimated number of cases Ebola cases for a mock city of 1,000,000 population in each of the three growth scenarios (delayed, Liberia-like, and fast response) four months (120 days) after introduction of a single case. The shaded boxes in the table highlight the numbers for the most likely response scenario given each country's economic category. Within each response scenario, we calculated low and high case count estimates based on number of cases that occur before detection and initiation of an effective response (lower: 10 cases; high: 100 cases). For example, consider the two extreme predictions in our model. We estimated that the Ebola outbreak in a mock city with a fast-response to the outbreak could have resulted in 287 Ebola cases if a total of 10 Ebola infections had occurred before the outbreak was detected and an effective response and control measures had been put in place (Table S4). On the upper end of the spectrum, the number of Ebola cases could have hypothetically reached about 17,992 cases if a total of 100 Ebola infections had occurred before the outbreak was detected and an effective response was put in place.

Table S4. Estimated number of Ebola cases in a mock city (population 1,000,000) four months (120 days) after introduction of a single case, for Liberia-like, delayed and fast scenarios of detection and initiation of an effective response (hospitalization and effective home isolation)*

	Response scenario		
	Delayed-response	Liberia-like	Fast-response
Lower (10 cases)*	917	367	139
High (100 cases)*	9,055	3,646	1,391

Notes: * The estimates shown in the table correspond to the output of EbolaResponse [1], using the parameters listed in Appendix 1, for each of the three response scenarios.

‡ Low and high case count estimates based on number of cases that occur before detection and initiation of an effective response (low: 10 cases; high: 100 cases).

There is substantial uncertainty in our estimates for a hypothetical broad-scale transmission of Ebola. We have no data to accurately predict the capacity of countries that were at high risk of Ebola transmission, as determined by air travel volume, of detecting and initiating an effective response to prevent or slow down an Ebola outbreak. Country's response capabilities are complex, and affected by a range of factors, as we discuss in section 2.3.2. The plausibility and uncertainty of our estimates may be well illustrated by the case of the Ebola outbreak in Nigeria.

Nigeria was quick to control an outbreak of Ebola that originated from a single infected traveler who flew from Liberia to Lagos in July 2014, which provides a good example for discussion [21-23]. In response to the rapid detection of Ebola, the Nigerian government, in collaboration with CDC and other partners, created an incident management system largely using staff from the Nigerian Polio Eradication Program and support from the Bill and Melinda Gates Foundation. Nigeria rapidly initiated a series of effective outbreak response measures, including training of healthcare workers, contact tracing, household visits, effective isolation of infectious patients, airport screening, and the creation of an Emergency Treatment Unit in two weeks. All these efforts by a well-staffed and prepared health workforce, which included support from the Polio Eradication Program, resulted in quickly controlling the Ebola outbreak. In contrast, the outbreak in Guinea took several weeks to be detected, and several more weeks before an effective response was put in place [24, 25]. The index case in Nigeria collapsed upon arrival to the airport in Lagos, which resulted in a very early detection of Ebola infection. That, plus the efforts from the local workforce of diversion of resources from the polio program resulted in a total of 19 cases.

For Nigeria (a lower-middle income country), if we considered a "Liberia-like" Ebola transmission and response and that only 10 cases of Ebola had occurred before outbreak detection and initiation of an effective response, our model results in an expected total of 627 cases of Ebola.

However, partially because Nigeria had public health capabilities from the Nigerian Polio Eradication Program and support from the Bill and Melinda Gates Foundation and thus quickly available resources and a trained health workforce, they put together a fast response. If we put these data into our model, assuming that only one Ebola case occurred before detection and effective response (as happened in reality) and that Nigeria had a “fast” response, we would expect a total of 28 Ebola cases, which is comparable with the 19 Ebola cases reported in Nigeria in a somewhat unique scenario. Our estimate is also comparable to a model based on the days before the intervention, assuming 12 exposed individuals from an index case [21].

2.3.2 Estimated number of Ebola cases using city-specific weights

To account for differences in living conditions between Monrovia, Liberia, and the cities used in the analyses, we performed two additional sets of analyses. We weighted the estimated number of Ebola cases that would occur using the ratio of: 1) each city’s population density (pop/sq. mile) to Monrovia’s population density, and 2) the ratio of the national proportion of the population living in slums in each country to the proportion of the population living in slums in Liberia (see main text for results).

We estimated the correlation coefficient between these two variables to check for co-linearity. Table S5 shows the Pearson’s R correlation coefficients for the following variables related to sociodemographic conditions within the cities selected for analysis: country population [19], total urban population living in slums [20], gross domestic product (GDP) per capita [26], World Bank economic category [18], population density [17], and proportion of the country’s population living in urban slums (compared to total population in the country). The country’s population and the number of people living in slums are highly correlated (r=0.96), as are the per capita GDP and the World Bank economic category. However, the correlations between all other pairs of variables are weak to moderate, ranging

from approximately r=-0.4 to r=0.5. Population density as a measure of overcrowding and the proportion of the population living in slums have an r = -0.10.

Table S5. Pearson's R correlation coefficients for measures of socioeconomic status, overcrowding and poverty for the 22 cities chosen for analysis

	Country population (total)*	Population in slums (total)†	GDP per capita (2013 US)‡	World Bank Economic category§	Population density (pop/sq.mi)	Pop. in slums (%)¶
Country population	1.00					
Population in slums	0.96	1.00				
GDP per capita	0.01	0.45	1.00			
World Bank economic category	0.35	0.53	0.73	1.00		
Population density	0.30	0.12	-0.26	-0.14	1.00	
Pop in slums (%)	-0.38	-0.39	-0.39	-0.35	-0.10	1.00

Notes: *Country population corresponds to estimates for year 2013 [19].
† Population in slums shows the estimated total urban population living in slums; the estimates were obtained at the country level from modelling [20].
‡ GDP per capita denotes the gross domestic product per person, values in 2015 US dollars [26].
§ Each year on July 1, the World Bank revises its analytical classification of the world's economies based on estimates of gross national income (GNI) per capita for the previous year [18].
¶ Pop. in slums denotes the proportion of the country's population living in urban slums (compared to total population in the country) in percentage [19, 20].

Last, for ease of comparison, Table S6 the estimated number of Ebola cases in each of 22 cities throughout Africa and Asia for three growth scenarios (delayed, Liberia-like, and fast response) (column A), scaled by the ratio of city's population density to the population density of Monrovia, Liberia (column B), and scaled by ratio of city's population in slums to the population in slums in Monrovia, Liberia (column C).

Table S6. Potential number of cases, given widespread Ebola transmission, for the cities with the higher travel volume to West Africa, considering (A) city population, (B) city population and population density compared to Monrovia/Liberia, and (C) city population and population in slums as compared to Monrovia/Liberia.

Economic category	Response	Location		A. Baseline, no weights		B. Weighted, pop. density*		C. Weighted, pop. in slums (%)*	
World Bank†	Scenario‡	City	Country	Low§	High§	Low§	High§	Low§	High§
Low-income	Delayed	Addis Ababa	Ethiopia	918	9,146	1,243	12,381	999	9,953
Low-income	Delayed	Banjul	Gambia	916	8,928	287	2,794	228	2,223
Low-income	Delayed	Bissau	Guinea-Bissau	916	8,922	1,128	10,986	430	4,191
Low-income	Delayed	Ouagadougou	Burkina Faso	918	9,138	937	9,324	1,152	11,468
Low-income	Delayed	Bamako	Mali	918	9,134	1,299	12,924	1,222	12,158
Low-income	Delayed	Lome	Togo	917	9,049	1,516	14,959	423	4,179
Lower-middle-inc.	Liberia-like	Nairobi	Kenya	319	1,563	477	2,339	422	2,066
Lower-middle-inc.	Liberia-like	Nouakchott	Mauritania	367	3,645	719	7,141	401	3,987
Lower-middle-inc.	Liberia-like	Dakar	Senegal	367	3,662	340	3,388	530	5,286
Lower-middle-inc.	Liberia-like	Mumbai	India	367	3,667	2,095	20,929	1,415	14,139
Lower-middle-inc.	Liberia-like	Delhi	India	367	3,667	779	7,783	1,728	17,271
Lower-middle-inc.	Liberia-like	Abidjan	Cote D'Ivoire	367	3,663	959	9,569	1,276	12,731
Lower-middle-inc.	Liberia-like	Accra	Ghana	367	3,658	277	2,762	402	4,008
Lower-middle-inc.	Liberia-like	Lagos	Nigeria	367	3,666	936	9,352	2,720	27,167
Lower-middle-inc.	Liberia-like	Casablanca	Morocco	367	3,662	764	7,623	239	2,380
Upper-middle-inc.	Fast	Istanbul	Turkey	139	1,394	239	2,399	403	4,039
Upper-middle-inc.	Fast	Beijing	China	139	1,394	134	1,347	906	9,083
Upper-middle-inc.	Fast	Guangzhou	China	139	1,394	147	1,470	501	5,020
Upper-middle-inc.	Fast	Wuhan	China	139	1,394	158	1,584	454	4,554
Upper-middle-inc.	Fast	Johannesburg	South Africa	139	1,394	79	797	453	4,547
Upper-middle-inc.	Fast	Beirut	Lebanon	139	1,393	80	805	281	2,813
Total				9,592	93,533	14,592	142,656	16,584	163,263

Notes: The results in column B of this table correspond to the results in Figure 2 of the main text.
* Adjustments based on population density and population in slums are included to account for the differences in living conditions between Monrovia, Liberia, and the cities used in the analyses, to account for sociodemographic factors in the risk of Ebola transmission [11, 27-32].
† Each year on July 1, the World Bank revises analytical classification of the world's economies based on estimates of gross national income (GNI) per capita for the previous year [18].
‡ The number of cases for each estimate are based in the economic category of the country. We assumed that low-income countries would have a delayed implementation of control measures to prevent Ebola spread, lower-middle income countries would have a Liberia-like implementation of control measures, and upper-middle and high-income would have a relatively fast implementation of control measures.
§ Low and high case count estimates based on number of cases that occur before detection and initiation of an effective response (low: 10 cases; high: 100 cases).

2.4 Potential broad-scale transmission of Ebola cases to major cities in within five selected countries

To illustrate the potential spread of Ebola within countries once a case had been imported into the main city, we selected five countries: Nigeria, Ethiopia, Kenya, South Africa and India. For each country we projected the number of EVD cases in major urban centers (populations of 100,000 or more). We chose these urban centers based on population size and travel access to the country's major urban area, either through air travel or that were located along major highways. The number of cases represents possible scenarios of intervention, and we used the same criteria as before: delayed transmission for lower income countries, Liberia-like transmission for lower-middle income countries, and fast transmission for upper income countries. Table S7 shows the estimated number of Ebola cases at four months (120 days) based on national resources and response scenarios, by city in selected countries (column A shows the baseline estimates, column B shows estimates weighted by population density). By design, the projected number of cases depends on the speed with which we assume that effective control measures are implemented. With our current assumptions, early detection of cases and rapid initiation of control measures is particularly important in the major cities of low-income countries (Ethiopia).

Table S7. Estimated number of Ebola cases at four months (120 days) based on national resources and response scenarios, in the case of intra-country spread for selected countries**.**

Economic category World Bank*	Country	Location City	City pop. (000s)†	Pop. dens. Ratio‡	A. Baseline, no weights		B. Weighted, pop. density	
					Low§	High§	Low§	High§
Low-income	Ethiopia	Addis Ababa	3238	1.35	918	9,146	1,243	12,381
Low-income	Ethiopia	Dire Dawa	342	1.14	915	8,811	1,039	10,010
Low-income	Ethiopia	Mek'ele	216	1.56	913	8,605	1,429	13,464
Low-income	Ethiopia	Nazret	220	1.30	913	8,615	1,186	11,194
Low-income	Ethiopia	Gondar	207	0.30	912	8,582	273	2,569
Lower-middle-inc.	Kenya	Nairobi	3915	1.50	367	3,662	549	5,481
Lower-middle-inc.	Kenya	Mombasa	1104	2.30	367	3,648	844	8,388
Lower-middle-inc.	Kenya	Kisumu	410	1.90	366	3,615	568	5,607
Lower-middle-inc.	Kenya	Nakuru	286	1.90	366	3,593	568	5,573
Lower-middle-inc.	Kenya	Eldoret	252	1.90	366	3,583	568	5,557
Lower-middle-inc.	Nigeria	Lagos	13123	2.55	367	3,666	936	9,352
Lower-middle-inc.	Nigeria	Ibadan	3160	1.20	367	3,661	439	4,383
Lower-middle-inc.	Nigeria	Abuja	2440	1.90	367	3,659	699	6,970
Lower-middle-inc.	Nigeria	Kano	3587	2.49	367	3,662	914	9,118
Lower-middle-inc.	Nigeria	Port Harcourt	2343	2.61	367	3,658	959	9,556
Lower-middle-inc.	Nigeria	Maidiguri	540	1.05	366	3,627	383	3,800
Lower-middle-inc.	India	Delhi	25703	2.12	367	3,667	779	7,783
Lower-middle-inc.	India	Jaipur	3073	1.45	367	3,661	532	5,305
Lower-middle-inc.	India	Lucknow	2901	1.73	367	3,657	637	6,344
Lower-middle-inc.	India	Kanpur	2920	2.59	367	3,660	949	9,461
Lower-middle-inc.	India	Agra	1746	2.64	367	3,655	969	9,647
Lower-middle-inc.	India	Mumbai	21043	5.71	367	3,667	2,095	20,929
Lower-middle-inc.	India	Ahmedabad	5633	2.11	367	3,664	774	7,727
Lower-middle-inc.	India	Pune	3124	2.07	367	3,661	759	7,571
Lower-middle-inc.	India	Hyderabad	8944	1.25	367	3,665	459	4,587
Lower-middle-inc.	India	Indore	1964	1.56	367	3,657	572	5,697
Upper-middle-inc.	South Africa	Johannesburg	9399	0.57	139	1,394	79	797
Upper-middle-inc.	South Africa	Durban	2901	0.56	139	1,393	78	787
Upper-middle-inc.	South Africa	Port Elizabeth	1179	0.55	139	1,392	77	767
Upper-middle-inc.	South Africa	Cape Town	3660	0.82	139	1,393	114	1,147
Upper-middle-inc.	South Africa	Bloemfontein	256	0.50	139	1,383	70	696
Upper-middle-inc.	Swaziland	Mbabane	66	0.11	139	1,.352	15	147
Upper-middle-inc.	Lesotho	Maseru	267	0.50	139	1,383	70	696
Total					13,247	130,097	21,624	213,488

Notes: The results in column B of this table correspond to the results in Figure 2 of the main text. The highlighted areas in the table represent most likely response scenario based on the country's economic category. Lower-middle-inc. denotes lower-middle-income country, and upper-middle-inc. denotes upper-middle-income country, as classified by the World Bank.
* Each year on July 1, the World Bank revises analytical classification of the world's economies based on estimates of gross national income (GNI) per capita for the previous year [18].
† Sources of data: CIA Factbook [33], Guangzhou population [34], Kenya 2009 Census [35], India 2011 census [36], South Africa 2011 Census [37], National Population Commission, Nigeria [38], Demographia [17].
‡ Ratio of city population density to population density of Monrovia, Liberia [17]. Population densities from Eldoret, Kisumu, and Nakuru were not available; the numbers reflect the average population density in Nairobi and Mombasa, Kenya.
§ Low and high case count estimates based on number of cases that occur before detection and initiation of an effective response (low: 10 cases; high: 100 cases).

3 Appendix S3. Travel data

Table S8 and Figure S3 show the air-traveler volumes from Sierra Leone, Guinea, and Liberia to the top 35 destination cities in the world, from July 2014 through December 2014. The table shows traveler volumes from each country to the main destination cities and the aggregate volume. We did not include travelers between Guinea, Liberia, and Sierra Leone. Data was obtained from BlueDot (Dr. Kamran Khan's database) which stratifies the arrivals by metro areas/cities [39]. Similar data was used to model the potential risk for international dissemination of EVD during the 2014-2015 outbreak in West Africa, and expected travelers infected with Ebola departing from Sierra Leone, Guinea, and Liberia [40]. We also included Addis Ababa (Ethiopia), Ouagadougou (Burkina Faso), and Wuhan (China) in the analysis, as these cities were the only top 25 destinations of travelers departing Guinea, Liberia, and Sierra Leone from September to December 2013 not included in the 2014 list. We excluded destination cities of high-income countries from our analysis.

Table S8. Air traveler volume from Sierra Leone, Guinea, and Liberia to the top 35 destination cities, July through December 2014

Destination Country	Destination City	City Code	Sierra Leone	Guinea	Liberia	Total
Ghana	Accra	ACC	5353	185	8295	13833
Morocco	Casablanca	CAS	1364	10250	1855	13469
France	Paris	PAR	650	11637	316	12603
Senegal	Dakar	DKR	3269	8957	360	12586
United Kingdom	London	LON	6942	909	1651	9502
Cote D'Ivoire	Abidjan	ABJ	2347	3971	2642	8960
Belgium	Brussels	BRU	2226	1418	5207	8851
United States	New York	NYC	1357	2484	2949	6790
Nigeria	Lagos	LOS	652	170	3384	4206
China	Beijing	BJS	886	956	1689	3531
Gambia	Banjul	BJL	3016	8	110	3134
United States	Washington	WAS	1547	296	1230	3073
Lebanon	Beirut	BEY	577	359	1757	2693
Mali	Bamako	BKO	27	2466	19	2512
Switzerland	Geneva	GVA	479	833	574	1886
Guinea-Bissau	Bissau	OXB	0	1833	0	1833
Kenya	Nairobi	NBO	589	27	904	1520
Togo	Lome	LFW	18	1082	92	1192
India	Mumbai	BOM	486	143	493	1122
India	Delhi	DEL	430	198	429	1057
United States	Minneapolis/St Paul	MSP	122	57	756	935
Turkey	Istanbul	IST	223	264	366	853
Canada	Montreal	YMQ	33	745	40	818
Netherlands	Amsterdam	AMS	397	296	117	810
United States	Atlanta	ATL	227	234	317	778
South Africa	Johannesburg	JNB	535	57	153	745
China	Guangzhou	CAN	111	465	121	697
Spain	Madrid	MAD	72	510	57	639
Mauritania	Nouakchott	NKC	0	634	0	634
Germany	Frankfurt	FRA	253	226	144	623
United Arab Emirates	Dubai	DXB	48	487	68	603
France	Lyon	LYS	40	526	28	594
United States	Boston	BOS	198	34	322	554
France	Toulouse	TLS	0	528	14	542

Notes: Data was obtained from BlueDot (Kamran Khan's database) which stratifies the arrivals by metro areas/cities.

Figure S3. Air traveler volume from Sierra Leone, Guinea, and Liberia to the top 35 city destinations, July through December 2014

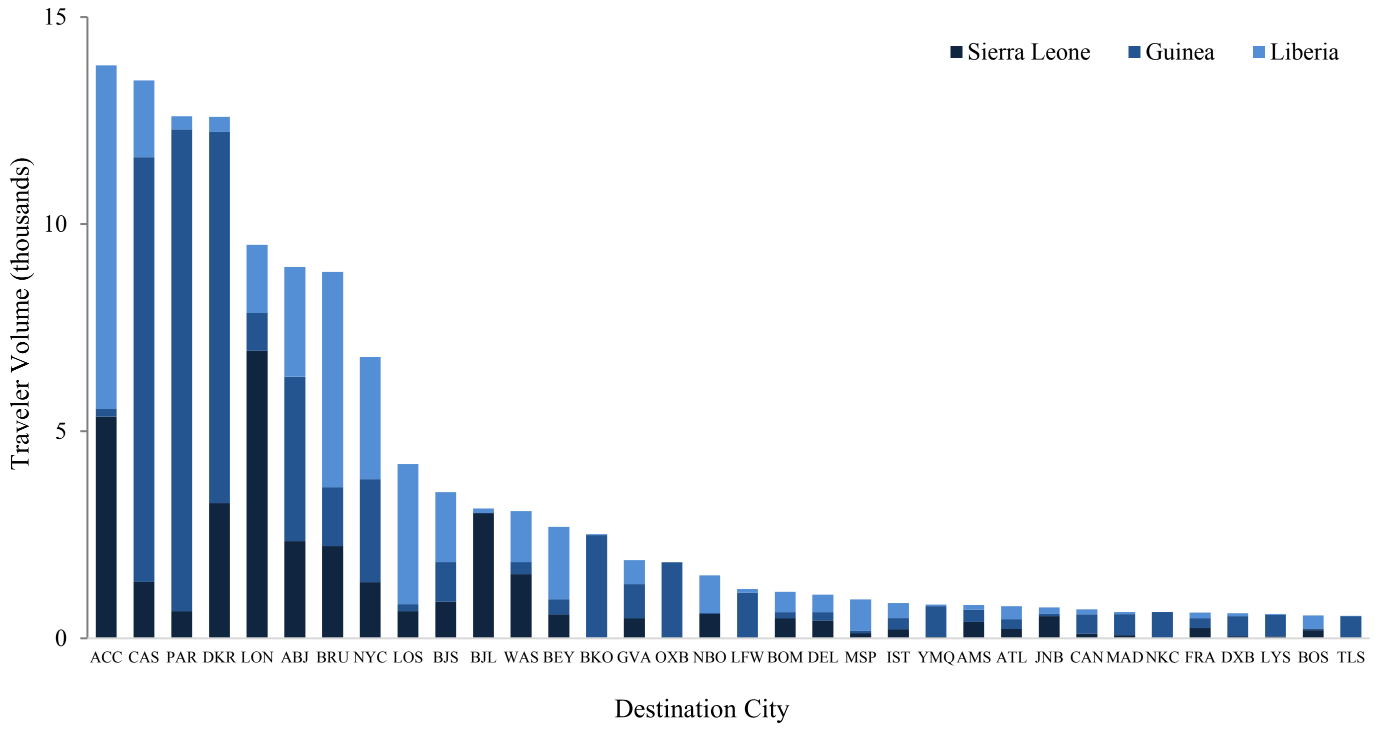

Notes: All city abbreviation codes can be found at: http://www.iata.org/publications/Pages/code-search.aspx

References for the supplementary material

1. Meltzer MI, Atkins CY, Santibanez S, Knust B, Petersen BW, Ervin ED, et al. Estimating the future number of cases in the Ebola epidemic—Liberia and Sierra Leone, 2014–2015. MMWR Morb Mortal Wkly Rep. 2014;63(Suppl 3):1-14.
2. Chowell G, Hengartner NW, Castillo-Chavez C, Fenimore PW, Hyman J. The basic reproductive number of Ebola and the effects of public health measures: the cases of Congo and Uganda. J Theor Biol. 2004;229(1):119-126.
3. Legrand J, Grais RF, Boelle P-Y, Valleron A-J, Flahault A. Understanding the dynamics of Ebola epidemics. Epidemiol Infect. 2007;135(4):610-621.
4. Eichner M, Dowell SF, Firese N. Incubation period of Ebola hemorrhagic virus subtype Zaire. Osong Public Health Res Perspect. 2011;2(1):3-7.
5. Aylward B, Barboza P, Bawo L, Bertherat E, Bilivogui P, Blake I, et al. Ebola virus disease in West Africa—the first 9 months of the epidemic and forward projections. N Engl J Med. 2014;371(16):1481-1495.
6. Chowell G, Nishiura H. Transmission dynamics and control of Ebola virus disease (EVD): a review. BMC Med. 2014;12(1):196.
7. Agua-Agum J, Ariyarajah A, Aylward B, Blake IM, Brennan R, Cori A, et al. West African Ebola epidemic after one year: slowing but not yet under control. N Engl J Med. 2015;372(6):584-587.
8. Faye O, Boëlle P-Y, Heleze E, Faye O, Loucoubar C, Magassouba NF, et al. Chains of transmission and control of Ebola virus disease in Conakry, Guinea, in 2014: an observational study. Lancet Infect Dis. 2015;15(3):320-326.
9. Van Kerkhove MD, Bento AI, Mills HL, Ferguson NM, Donnelly CA. A review of epidemiological parameters from Ebola outbreaks to inform early public health decision-making. Sci Data. 2015;2:150019.
10. Lekone PE, Finkenstädt BF. Statistical inference in a stochastic epidemic SEIR model with control intervention: Ebola as a case study. Biometrics. 2006;62(4):1170-1177.
11. Hewlett BS, Amola RP. Cultural contexts of Ebola in northern Uganda. Emerg Infect Dis. 2003;9(10):1242.
12. Drazen JM, Kanapathipillai R, Campion EW, Rubin EJ, Hammer SM, Morrissey S, et al. Ebola and quarantine. N Engl J Med. 2014;371(21):2029-2030.
13. Centers for Disease Control and Prevention. Review of human-to-human transmission of Ebola virus Atlanta, GA2016; Accessed June 20 2017. Available from: https://www.cdc.gov/vhf/ebola/transmission/human-transmission.html#17.
14. Centers for Disease Control and Prevention. 2014 Ebola Outbreak in West Africa - Case Counts Atlanta, GA: CDC; 2016; Accessed 10 August 2016. Available from: https://www.cdc.gov/vhf/ebola/outbreaks/2014-west-africa/case-counts.html.
15. Townsend JP, Skrip LA, Galvani AP. Impact of bed capacity on spatiotemporal shifts in Ebola transmission. Proc Natl Acad Sci. 2015;112(46):14125-14126.

16. Nouvellet P, Garske T, Mills HL, Nedjati-Gilani G, Hinsley W, Blake IM, et al. The role of rapid diagnostics in managing Ebola epidemics. Nature. 2015;528(7580):S109-S116.
17. Demographia. World urban areas. 11th annual edition, January 2015; Accessed January 2016. Available from: http://www.demographia.com/db-worldua.pdf.
18. World Bank. Data. Updated income classifications: World Bank; 2015; Accessed December 2015. Available from: http://data.worldbank.org/news/2015-country-classifications.
19. World Health Organization. World Health Statistics Report 2015; Accessed December 2015. Available from: http://www.who.int/gho/publications/world_health_statistics/2015/en/.
20. United Nations. Slum population in urban areas (thousands). Millenium Development Goal Indicators: United Nations Statistics Division; 2015; Accessed December 2015. Available from: http://mdgs.un.org/unsd/mdg/SeriesDetail.aspx?srid=711.
21. Fasina FO, Shittu A, Lazarus D, Tomori O, Simonsen L, Viboud C, et al. Transmission dynamics and control of Ebola virus disease outbreak in Nigeria, July to September 2014. Euro Surveill. 2014;19(40):20920.
22. Shuaib F, Gunnala R, Musa EO, Mahoney FJ, Oguntimehin O, Nguku PM, et al. Ebola virus disease outbreak—Nigeria, July–September 2014. MMWR Morb Mortal Wkly Rep. 2014;63(39):867-872.
23. Frieden TR, Damon IK. Ebola in West Africa—CDC's role in epidemic detection, control, and prevention. Emerg Infect Dis. 2015;21(11):1897.
24. Baize S, Pannetier D, Oestereich L, Rieger T, Koivogui L, Magassouba NF, et al. Emergence of Zaire Ebola virus disease in Guinea. N Engl J Med. 2014;371(15):1418-1425.
25. Dixon MG, Schafer IJ. Ebola viral disease outbreak—West Africa, 2014. MMWR Morb Mortal Wkly Rep. 2014;63(25):548-551.
26. World Bank. World Bank Open Data 2015; Accessed December 2015. Available from: http://data.worldbank.org/.
27. Alexander KA, Sanderson CE, Marathe M, Lewis BL, Rivers CM, Shaman J, et al. What factors might have led to the emergence of Ebola in West Africa? PLoS Negl Trop Dis. 2015;9(6):e0003652.
28. Chan M. Ebola virus disease in West Africa—no early end to the outbreak. N Engl J Med. 2014;371(13):1183-1185.
29. Nielsen CF, Kidd S, Sillah A, Davis E, Mermin J, Kilmarx PH. Improving burial practices and cemetery management during an Ebola virus disease epidemic—Sierra Leone, 2014. MMWR Morb Mortal Wkly Rep. 2015;64(1):20-27.
30. Piot P. Ebola's perfect storm. Science. 2014;345(6202):1221-1221.
31. Bausch DG, Schwarz L. Outbreak of Ebola virus disease in Guinea: where ecology meets economy. PLoS Negl Trop Dis. 2014;8(7):e3056.
32. Buseh AG, Stevens PE, Bromberg M, Kelber ST. The Ebola epidemic in West Africa: challenges, opportunities, and policy priority areas. Nurs Outlook. 2015;63(1):30-40.

33. CIA. The World Factbook: Central Intelligence Agency; 2015; Accessed December 2015. Available from: https://www.cia.gov/library/publications/the-world-factbook/fields/2219.html.
34. Statistics Bureau of Guangzhou. Statistics Bureau of Guangzhou Municipality: The People`s Government of Guangzhou Municipality,; 2015; Accessed December 2015. Available from: http://english.gz.gov.cn/gzgoven/s3709/201008/787559.shtml.
35. Kenya Open Data. 2009 Census Vol 1 Table 3 Rural and Urban Population 2015; Accessed December 2015. Available from: https://www.opendata.go.ke/Population/2009-Census-Vol-1-Table-3-Rural-and-Urban-Populati/e7c7-w67t/1.
36. Census India. Provisional population totals, Census of India 2011 2015; Accessed December 2015. Available from: http://censusindia.gov.in/2011-prov-results/paper2/data_files/India2/Table_3_PR_UA_Citiees_1Lakh_and_Above.pdf.
37. Statistics South Africa. Census 2011 Community Profile Databases 2015; Accessed December 2015. Available from: http://www.statssa.gov.za/.
38. National Population Comission Nigeria. Population distribution by sex, state, lgas, and senatorial district: 2006 census priority tables 2015; Accessed December 2015. Available from: http://www.population.gov.ng/index.php/publications/140-population-distribution-by-sex-state-lgas-and-senatorial-district-2006-census-priority-tables-vol-3.
39. BlueDot. Transportation networks 2015; Accessed December 2015. Available from: http://bluedot.global/.
40. Bogoch II, Creatore MI, Cetron MS, Brownstein JS, Pesik N, Miniota J, et al. Assessment of the potential for international dissemination of Ebola virus via commercial air travel during the 2014 west African outbreak. Lancet. 2015;385(9962):29-35.
